# Building capacity for network meta-analysis in Sub-Saharan Africa: reflections and future direction

**DOI:** 10.1186/s13643-023-02418-8

**Published:** 2024-01-02

**Authors:** Anke Rohwer, Veranyuy Ngah, Dimitris Mavridis, Taryn Young, Michael McCaul

**Affiliations:** 1 Centre for Evidence-based Health Care, Division of Epidemiology and Biostatistics, Department of Global Health, Faculty of Medicine and Health Sciences, Stellenbosch University, Cape Town, South Africa; 2 Department of Primary Education, University of Ioannina, Ioannina, Greece; 3 South African GRADE Network, Stellenbosch University, Cape Town, South Africa

**Keywords:** Systematic reviews, Capacity development, Network meta-analysis, Low- to middle-income countries, Evidence-based healthcare

## Abstract

Robust, relevant, comprehensive, and up-to-date evidence syntheses are the cornerstone for evidence-informed healthcare decisions. When considering multiple treatment options, network meta-analysis (NMA) systematic reviews play a key role in informing impactful decisions and clinical practice guidelines. However, the capacity and literacy to conduct NMA systematic reviews and interpret its results remains out of reach for many clinicians and review authors, especially in low-to-middle-income countries. Despite ample resources and guides, NMA capacity and training opportunities remain limited to non-existent in Sub-Saharan Africa. Towards solutions and strengthening evidence synthesis and NMA capacity in the Sub-Saharan African region, we describe and reflect on two courses that build NMA capacity and aim to address NMA literacy in Sub-Saharan Africa.

The Primer in NMA systematic reviews aimed for participants to be able to find, appraise, interpret, and consider the use of NMA SRs of intervention effects. It is a 6-week online course for clinicians, policy-makers, and researchers wanting to learn more about using NMA systematic reviews. The Global NMA Masterclass workshop aimed for participants to be able to understand and apply pairwise and NMA in STATA and R, evaluate NMA assumptions and confidence in NMA results, and appropriately report NMA results. This course was offered over 5 weeks to clinicians, biostatisticians, and researchers with basic knowledge of epidemiology and biostatics. Although the bulk of learning occurred through self-study, we had weekly, synchronous question-and-answer sessions for both courses. Using relevant examples throughout the courses helped to enable an authentic learning environment.

This was the first NMA training developed in Africa for Africa. Development of the courses was a collaborative effort from a multi-disciplinary team. Both NMA courses were well received and attended by a diverse group of participants spread across Sub-Saharan African countries. Participants felt the courses were applicable to their setting. Although most participants appreciated the benefits of online learning, we also experienced some challenges. There is great potential to conduct NMA systematic reviews in Sub-Saharan Africa. The NMA Primer and NMA workshop can play an essential role in expanding and developing NMA SR capacity and literacy in SSA.

## Background

Robust, relevant, and up-to-date evidence syntheses are the cornerstone for evidence-informed healthcare decisions, impactful policy, and clinical practice. Generation, access to, and application of trustworthy evidence is paramount globally, but especially so in sub-Saharan Africa (SSA) where the burden of disease is high and resources are scarce [1]. The effects of pandemics, and infodemics — the rapid spread and generation of accurate and inaccurate information — are felt more in low-resource settings such as SSA because of the fragile healthcare systems and infrastructure [2]. This is likely to impact people living with HIV and other infectious diseases. In addition, non-communicable diseases associated with HIV/AIDS, including hypertension, heart disease, diabetes, and cancer, continue to grow as the survival of HIV-infected people continues to improve with new effective treatments [3, 4]. These current epidemics and potential future unknown epidemics and infodemics in SSA demand that institutions continue to invest not only in local research capacity to conduct research, but also in advancing capacity to use research, especially evidence synthesis, in healthcare decision making.

There is a dire need for data-driven innovations and interventions supported by scientific rigor through unbiased and well-controlled experimental design, methodology, analysis, interpretation, and reporting of results. Systematic reviews (SR) (with or without meta-analysis) provide the most unbiased picture of the evidence to inform healthcare decisions and clinical practice guidelines. The most common technique of meta-analysis in a SR is to compare only one intervention to another (i.e., pair-wise meta-analysis), which has limited use and applicability, as often multiple treatment options exist for a single condition. Clinicians or decision makers need to consider all available treatment options to make informed healthcare choices [5].

Network meta-analysis (NMA) solves this problem, by comparing multiple treatment options for a specific condition in a SR, combining both direct and indirect evidence, in a network of trials. NMA allows decision makers (e.g., in clinical practice guidelines) to answer questions, when current trials do not exist, via indirect evidence. Additionally, NMA allows reviewers to rank interventions which is key in conducting SRs and making healthcare decisions. Network meta-analysis use is increasing worldwide and is emerging in World Health Organization (WHO) guidelines and in Cochrane and spearheading the “new standard” in evidence synthesis for COVID-19 interventions globally (www.covid-nma.com) [6], and in HIV [7].

In the continued response to the COVID-19 pandemic and future pandemics, the low-to-middle-income countries (LMICs) evidence synthesis community represents an “untapped well” of expertise in evidence synthesis and knowledge translation, with the potential to prominently impact evidence-informed decision making across the world [8]. However, capacity (to do) and literacy (to use) in NMA, which is essential for making complex healthcare decisions, are limited. This is especially prominent in Africa where few African authors are leading or publishing NMA SRs and are thus extremely underrepresented compared to developed regions and countries [9, 10]. Although great progress and rapid growth have been made in “standard” SRs in Africa regarding research capacity, support initiatives and access to information [11], NMA capacity in the region is yet to catch up. Furthermore, to respond to the increasingly complex health decisions brought by COVID-19 and in people living with HIV/AIDS, decision makers need to be able to find, use, appraise, and interpret complex evidence from NMA SRs for decision making. Additionally, the “untapped well” of HIV/AIDS researchers and biostatisticians in SSA need to be equipped in conducting NMA in SRs to inform healthcare decisions that are efficient and both useful to decision makers and guideline developers, such as WHO and professional societies.

Various resources exist for conducting NMAs, ranging from technical support documents, to reporting guidelines, to handbooks [12]. However, despite this range of resources, NMA is still considered a “black box” by many, including novice and experienced SR authors. Network meta-analysis is also still unfamiliar to many biostatisticians. This may be due to analytical complexity of this method, lack of local expertise in NMA methods and training opportunities, as well as the lack of a clear guide to support the key decisions required to successfully conduct a NMA SR. Furthermore, due to variation in quality reporting, decision makers and guideline development groups face additional biostatistical literacy challenges when reading and interpreting such reviews informing public health decisions. In SSA, formal training SRs and pair-wise meta-analysis are well established [13]; however, in NMA, training is limited or close to non-existent. Access to these courses beyond the region is often prohibitively costly due to decreased buying power and currencies, excluding international travel and accommodation. Towards solutions and strengthening evidence synthesis and NMA capacity in the region, we describe two courses that build NMA capacity and aim to address NMA literacy in Sub-Saharan Africa.

## Network meta-analysis training programs

We developed two courses on NMA, the *Primer in NMA Systematic Reviews* (hereafter referred to as NMA Primer) and a *Global NMA Masterclass workshop* (hereafter referred to as NMA workshop) (Table 1). The Primer focuses on finding, reading, and understanding NMA SRs (i.e., using NMA reviews), while the NMA workshop focuses on conducting NMA using both freely available (R) and commercial statistical software (Stata). The two courses therefore differ in their primary aim but complement each other very well. Both these courses are the first of their kind on NMA SRs in the region.

**Table 1:** Summary of NMA capacity-building initiatives

Name of course	Primer in NMA Systematic Reviews	Global NMA Masterclass workshop
Aim	For participants to be able to find, appraise, interpret, and consider the use of NMA SRs of intervention effects.	For participants to be able to understand and apply pairwise and NMA in STATA and R, evaluate NMA assumptions and confidence in NMA results, and appropriately report NMA results.
Format	Online	Online
Duration	8 weeks	5 weeks
Learning tools	- Text-based lessons - Readings - Videoclips - Asynchronous discussion forum. - Synchronous question and answer session with facilitators. - Self-directed exercises - Self-assessments	- Recorded lecture on core topics. - Practical tasks in STATA or R. - Demonstration of implementing the task in STATA or R. - Asynchronous discussion forum. - Synchronous weekly sessions with facilitators.
Participants	Ethiopia, Nigeria, South Africa, Uganda, Zambia, Zimbabwe, Australia, UK	South Africa, Kenya, Malawi, Zimbabwe, Nigeria, Uganda
Entry requirements	None	Basic knowledge of biostatistics and epidemiology Experience in SRs or attending Primer in NMA SRs advantageous Experience in doing a pair-wise meta-analysis

**Fig. 1:**
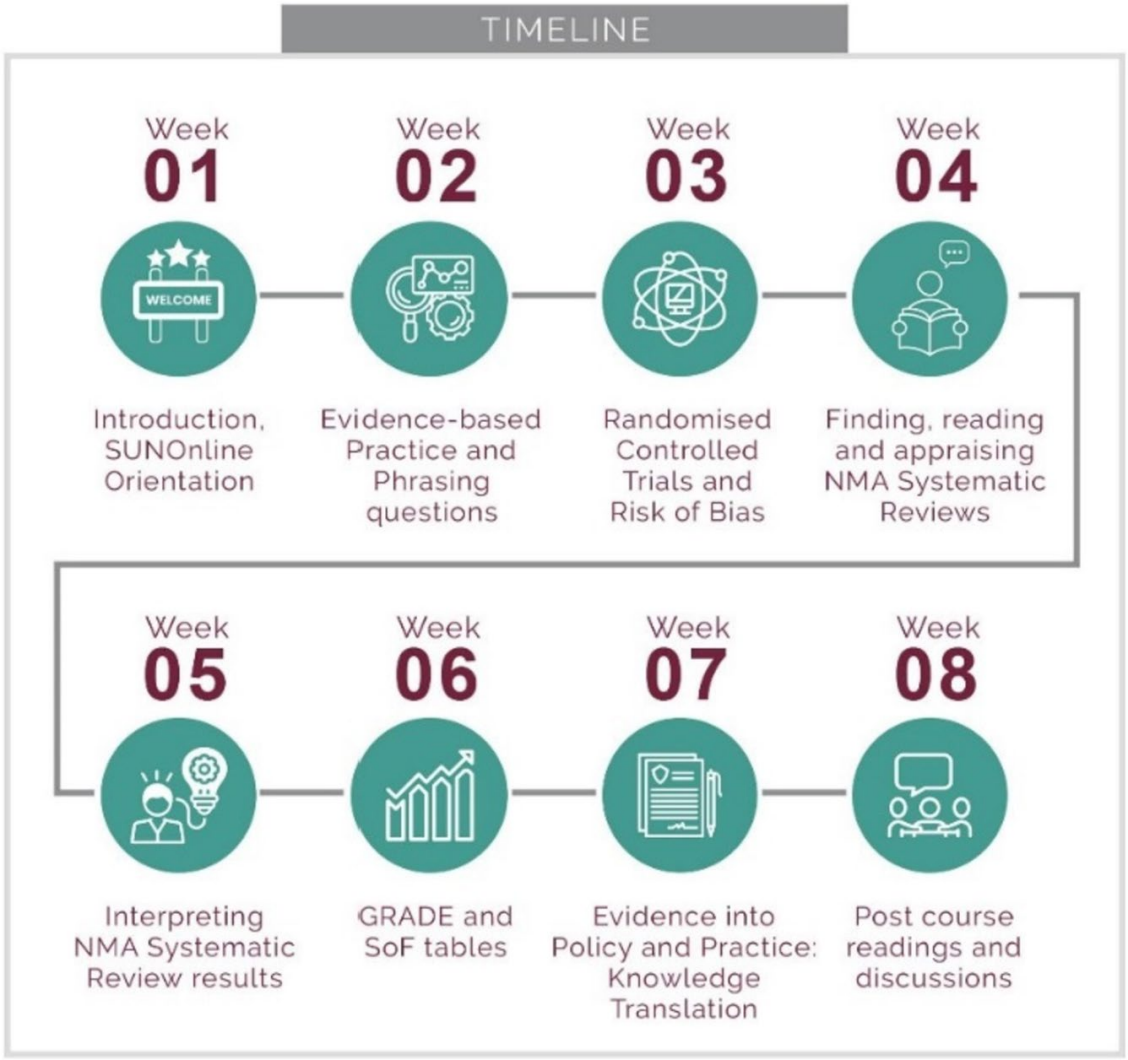
Outline of Primer in NMA Systematic Reviews

The aim of the NMA Primer was for participants to be able to find, appraise, interpret, and consider the use of NMA SRs of intervention effects. This course was developed by adapting and expanding the existing online *Primer in Systematic Reviews* course [14]. This course was originally developed and first offered in 2012 as a 4-day face-to-face short course, aiming for participants to understand, appraise, and use SRs of effects of interventions. Subsequently, a 6-week online version of the course was developed as an official short course at Stellenbosch University, South Africa. It was first offered in 2016 and has been running twice a year since then. A multidisciplinary team, consisting of SR and NMA experts (AR, MM, DM), a technical editor (KM), and a learning management systems expert (MC) collaborated to adapt the content and format of the original course. The NMA Primer runs over eight weeks in a modular and linear way (Fig. 1). It is a fully online course comprising various learning opportunities (Table 1). We integrated clinical examples relevant to SSA (second-line treatment for HIV and COVID-19) [15, 16], as well as self-directed exercises based on these examples throughout the lessons. Twenty-five participants completed the NMA Primer, including post-graduate students, clinicians working in the field of HIV, as well as decision-makers in the field of HIV and COVID-19. Participants were based in various countries from SSA, including Nigeria, Zimbabwe, South Africa, Ethiopia, Uganda, and Zambia. At the end of the course, participants completed a short evaluation and received a certificate of attendance from Stellenbosch University.

**Fig. 2:**
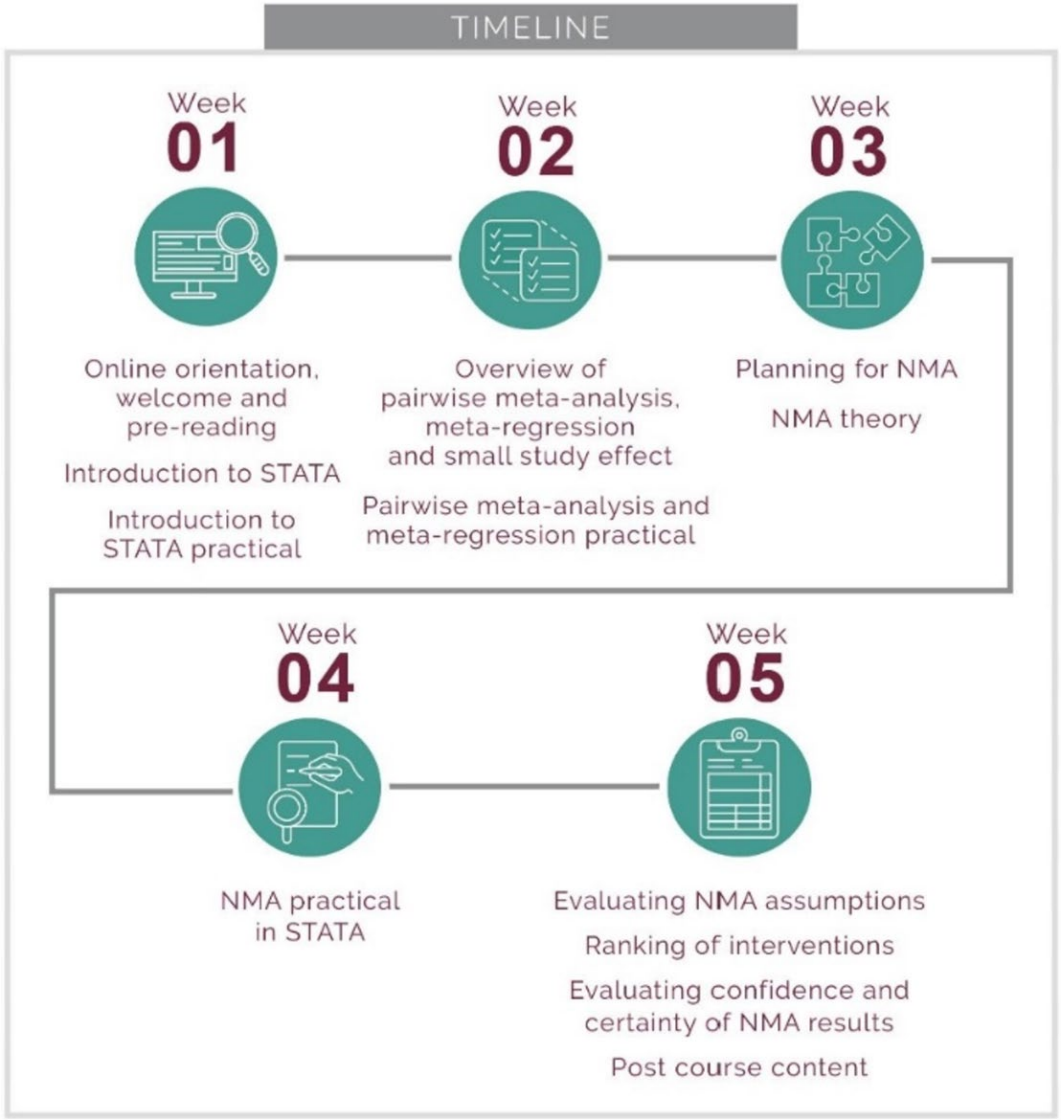
Outline of the NMA workshop

The NMA workshop aims to build regional capacity in conducting SRs using NMA in STATA and R. This was planned as a 4-day face-to-face workshop, but due to the impact of the COVID-19 pandemic, we reorganized the workshop to be fully online over a 5-week period. After the workshop, participants should be able to understand and apply pairwise and network meta-analysis in STATA and R, evaluate NMA assumptions and confidence in NMA results, and appropriately report NMA results. Weeks were structured in a modular and linear way (Fig. 2) and comprised a recorded lecture from a NMA expert (DM) on a core topic, practical tasks in STATA or R, as well as a demonstration of implementing the task in STATA or R, supplemented with discussions (Table 1) and increasing relevance through use of examples linked to HIV and COVID-19. Eighteen participants completed the NMA workshop, including clinicians, biostatistics students, and South African National Department of Health decision makers, all working within the field of HIV and/or COVID-19. Participants were based in South Africa, Malawi, Ethiopia, Kenya, Uganda, Zimbabwe, Tanzania, and Nigeria. Participants needed to have basic knowledge of epidemiology and biostatistics, while having previously conducted a SR or having attended the Primer in NMA SRs was advantageous. Post-COVID-19, we offered the NMA workshop in a face-to-face format over 3 days to biostatistics and clinical epidemiology students, faculty staff, and researchers in Cape Town, South Africa. After the workshop, participants completed a short evaluation form and received certificates of attendance.

## Reflections and future directions

To our knowledge, the two NMA courses were the first courses developed in Africa for Africa. We identified the need to build capacity for NMA in SSA, as researchers, clinicians, and policymakers in the region require the capacity to use, and in some cases conduct NMA SRs, and yet do not always have the resources and opportunities to attend courses offered in the global North. The two courses were well suited to meet these needs.

We took a multi-pronged approach to develop the NMA courses. First, we took stock of existing courses on SRs and NMA in the region, to ensure that we did not re-invent the wheel. Furthermore, we built on existing training initiatives to maximize available resources and acknowledge that a fair amount of previous work, commitment, and experience enabled us to develop both courses. Developing the courses was a collaborative effort, where each team member’s individual skills and expertise complemented each other very well. This proved to be pivotal in the successful development and implementation of these courses. Lastly, according to the best available evidence on teaching evidence-based practice [17, 18], our courses were multi-faceted, used a blended approach, and included examples relevant to the target audience to enhance the authenticity of learning. We made use of a variety of active learning strategies to promote deep learning and included exercises and self-assessments to provide an opportunity for revision.

A diverse group of participants attended the Primer and the workshop, with a few attending both courses. Participants were spread across SSA and had varying background knowledge of SRs. Entry requirements for the workshop were much stricter than for the Primer, as conducting a NMA SR required some basic knowledge of epidemiology and biostatistics. This was a limitation for some participants, as we did not allocate time to recap basic epidemiological or biostatistical principles during the workshop. Although having conducted SRs and doing pair-wise meta-analysis previously was advantageous, few participants actually had that experience.

A big advantage of the online courses was that learners were able to go through the material at their own pace, anytime, anywhere. On the other hand, some learners might have had limited motivation and commitment to engage in self-study. We found that some participants were more active and engaging than others, but this is a common challenge with online, adult learning. Furthermore, internet connectivity remains a challenge in SSA, and it affected the ability to participate in synchronous sessions.

The Primer was developed for busy clinicians and policymakers with a weekly time commitment of 2 to 4 h. The workshop was more intense and required participants to spend about 4 to 5 h per week, although this might not have been enough time to work through all the exercises for some participants. Feedback from participants confirmed that it was not always easy to keep up with the content and some wanted more time to go through content. However, as both courses have a start and end date, it was important for them to maintain their pace and the dynamics of the courses. Furthermore, both NMA courses covered complex concepts, and it was essential that participants go through the content as required for each week. We encouraged participants to continue with the course even if they were still catching up. However, some attrition was inevitable, despite allocating an additional week to access the learning material after the course had ended.

Although we included a weekly, synchronous question and answer session to clarify complex concepts, these were not always well attended. Participants did not engage in a lot of conversations on the asynchronous discussion forums. A few participants that attended the NMA workshop felt that these concepts would be easier to understand in a face-to-face workshop. Key to understanding complex concepts is active, in-depth learning, which can be achieved with online or in-person learning. However, it is important that facilitators encourage and provide a space for active learning so that participants engage with the content in a meaningful way. We tried to do this through asynchronous and synchronous discussions, but for some engaging with peers and facilitators might come more naturally in a face-to-face format. This was evident when we offered the NMA workshop in-person, where conversations were lively, and participants actively engaged with peers and facilitators.

This was an exciting endeavor that was well received by participants. Informal feedback remained positive, with participants acknowledging the added value in their daily clinical load, research, and future studies. Many appreciated the flexibility of online learning and ease of the learning platform.

There is great potential to conduct NMA reviews across SSA. Although we did not actively follow-up with participants after the courses, we know that at least one of the participants has subsequently embarked on a Cochrane review and NMA [19]. Formal assessment of the acquisition and application of new knowledge and skills will be useful.

## Conclusion

The NMA Primer and NMA workshop can play an essential role in expanding and developing NMA SR capacity and literacy in SSA. Further collaboration and harnessing the benefits of an online course while being cognizant of the challenges will be critical for future courses. Mentorship will be key to increase the production of NMA reviews by African authors.

## Data Availability

Not applicable

## Abbreviations

NMA: Network meta-analysis
SR: Systematic review
HIV/AIDS: Human immunodeficiency virus/ acquired immunodeficiency syndrome
SSA: Sub-Saharan Africa
